# Selenium-Enriched Polysaccharides from *Lentinula edodes* Mycelium: Biosynthesis, Chemical Characterisation, and Assessment of Antioxidant Properties

**DOI:** 10.3390/polym17060719

**Published:** 2025-03-09

**Authors:** Eliza Malinowska, Grzegorz Łapienis, Agnieszka Szczepańska, Jadwiga Turło

**Affiliations:** 1 Department of Drug Technology and Pharmaceutical Biotechnology, Medical University of Warsaw, 1 Banacha Str., 02-097 Warszawa, Poland; agnieszka.szczepanska@wum.edu.pl (A.S.); jadwiga.turlo@wum.edu.pl (J.T.); 2 Department of Functional Polymers and Polymeric Materials, Centre of Molecular and Macromolecular Studies, Polish Academy of Sciences, 112 Sienkiewicza Str., 90-363 Łódź, Poland; grzegorz.lapienis@cbmm.lodz.pl

**Keywords:** selenium-enriched polysaccharide, antioxidant activity, *Lentinula edodes* mycelium, bioactive compounds

## Abstract

Selenium–polysaccharides possess antioxidant properties, making them promising materials for functional foods, pharmaceuticals, and clinical applications. This study examines the incorporation of selenium into polysaccharides via mycelial biosynthesis and its effects on structure and antioxidant activity. Polysaccharides obtained from *Lentinula edodes*-submerged cultures grown in Se-supplemented and non-supplemented media were analysed for Se content (RP-HPLC/FLD), structure (FT-IR, HPLC, and HPGPC-ELSD), and antioxidant activity (DPPH scavenging, reducing power, and Fe^2+^ chelation). Two low-molecular-weight Se–heteropolysaccharides (Se-FE-1.1 and Se-FE-1.2) containing ~80 and 125 µg/g Se were isolated, primarily composed of glucose, mannose, and galactose with β-glycosidic linkages. Se incorporation into polysaccharides selectively enhanced their antioxidant activity in the DPPH radical scavenging assay, with minimal effects observed in iron chelation and reducing power assays. Crude Se–polysaccharides displayed the highest antioxidant activity, suggesting an additional contribution from protein components. Our findings demonstrate that Se is effectively incorporated into polysaccharides, altering monosaccharide composition while preserving glycosidic linkages. The selective enhancement of radical scavenging suggests that selenium plays a specific role in antioxidant activity, primarily influencing radical scavenging mechanisms rather than interactions with metal ions. Further research is needed to clarify the mechanisms of selenium incorporation, the nature of its bonding within the polysaccharide molecule, and its impact on biological activity.

## 1. Introduction

The search for new antioxidant compounds has gained importance due to their potential health benefits and role in functional foods. Antioxidants help mitigate the harmful effects of free radicals, which contribute to chronic diseases like cardiovascular issues, cancers, and neurodegenerative disorders [1]. Incorporating antioxidants into functional foods can significantly enhance health and support therapeutic interventions.

Current antioxidants face limitations such as low bioavailability, reduced stability, and potential adverse effects from long-term use [2]. Therefore, there is growing interest in naturally derived compounds. Edible mushrooms, rich in bioactive antioxidants, have emerged as promising candidates for functional foods, aligning with consumer demand for natural ingredients.

One promising approach involves modifying polysaccharides biosynthesised by fungi by incorporating trace elements such as selenium (Se), a micronutrient with potent antioxidant properties. Se can significantly impact the structure and function of polysaccharides, leading to the formation of new compounds known as Se–polysaccharides, which may offer enhanced health benefits and novel therapeutic potential because of the synergistic effects of Se and polysaccharides. As organic forms of Se, Se–polysaccharides have garnered increasing attention for their superior bioavailability and reduced toxicity compared with inorganic Se forms. Studies indicate that biosynthetically Se-enriched polysaccharides isolated from fungi, along with synthetic Se-containing polysaccharide derivatives, can exhibit bioactivities that are significantly greater than those of Se or polysaccharides alone, including an enhanced ability to neutralise reactive oxygen species [3,4,5,6,7,8,9,10,11,12]. Moreover, Se modification improves the physicochemical properties of polysaccharides, increasing molecular weight, reducing particle size, and enhancing stability in solution. It also induces conformational changes in the polysaccharide chains, further augmenting their antioxidant potential [13].

*Lentinula edodes* (shiitake mushroom) is renowned for bioactive polysaccharides, like lentinan, which exhibit immunomodulatory and anticancer properties. Our previous studies demonstrated that *L. edodes* mycelium grown in a Se-enriched medium incorporates Se into its polysaccharides, leading to structural modifications and enhanced biological activity. The resulting Se–polysaccharides exhibited potent antioxidant and selective immunosuppressive effects [14,15,16].

The current study investigates Se-enriched polysaccharides from submerged *L. edodes* cultures, distinct from those previously examined, and compares them with those obtained without Se supplementation to evaluate their antioxidant potential.

Our main objectives were as follows:

- To conduct a structural analysis, including assessment of the Se content, molecular weight, monosaccharide composition, and anomeric configuration of glycosidic bonds;
- To assess the antioxidant activity of the compounds through DPPH scavenging, reducing power, and ferrous ion chelation;
- To determine whether and how Se incorporation and the structural features of the polysaccharides affect their ability to neutralise free radicals.

This study will complement our understanding of Se-enriched polysaccharides and their antioxidant potential, providing insights into their potential applications in functional foods and therapeutic interventions.

## 2. Materials and Methods

### 2.1. Microorganism and Cultivation Media

The *L. edodes* (Berk.) Pegler reference strain was purchased from the American Type Culture Collection. Stock cultures (ATCC 48,085; Manassas, VA, USA) were maintained on slants of malt agar at 4 °C and transferred to a fresh slant every 3 months. Liquid cultivation of *L. edodes* was carried out in 500 mL Erlenmeyer flasks containing 200 mL of Sabouraud Dextrose Broth medium (Biocorp Polska, Warsaw, Poland), consisting of 20 g/L glucose and 10 g/L peptone (meat and casein), at 26 °C, on a rotary shaker (New Brunswick Scientific, Edison, NJ, USA) set to 120 rpm, for 14 days. The seed cultures obtained were used for inoculation. Se-fortified mycelia were cultivated under submerged conditions in a 10 L fermenter (BioTec FL 110, Stockholm, Sweden) containing nutrient medium with the following composition: glucose, 5.0 g/L; yeast extract, 10.0 g/L; corn steep liquor, 10.0 g/L; soy peptone, 15 g/L; KH_2_PO_4_, 1.0 g/L; and Na_2_SeO_3_, 30 mg/L. Control medium was prepared without the addition of sodium selenite. Mycelium was harvested by filtration, washed three times with distilled water, and freeze-dried.

### 2.2. Extraction and Fractionation of Se-Enriched Polysaccharides

#### 2.2.1. Extraction Procedure

**Figure 1:**
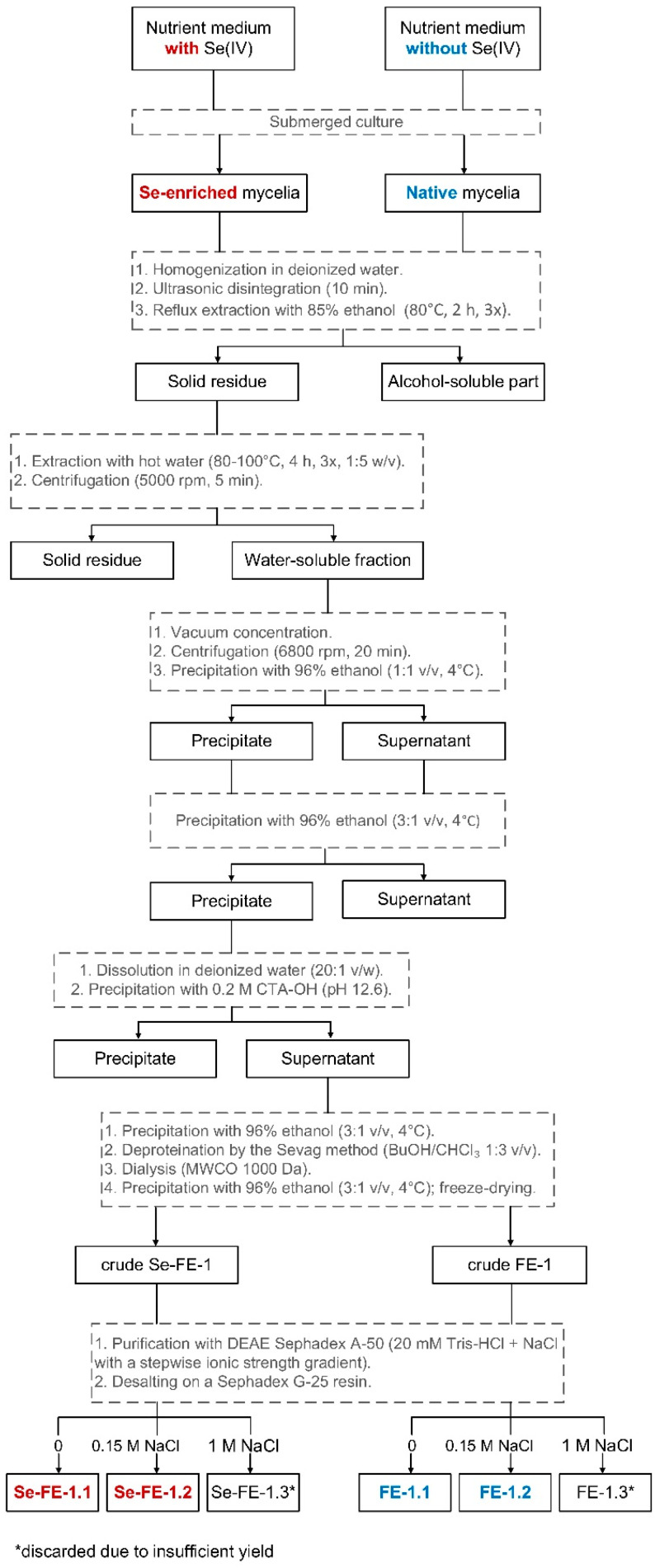
Schematic representation of the isolation and purification process for Se-enriched polysaccharides and their non-selenised analogues. *discarded due to insufficient yield.

A schematic representation of the extraction and purification procedure for polysaccharides is shown in Figure 1.

The Se-enriched biomass obtained from several bioreactors was soaked in deionised water, blended (T 25 digital ULTRA-TURRAX, IKA, Warsaw, Poland), and initially disintegrated in an IS-2 ultrasonic cleaning device (InterSonic, Olsztyn, Poland) for 10 min. The mycelium was then extracted three times with 85% ethanol (80 °C, 6 h) to remove substances of low-to-medium polarity (e.g., lipids and cell membrane components). After discarding the ethanol extracts, the defatted biomass was suspended in deionised water at a weight ratio of 30:1 and subjected to three rounds of hot water extraction with continuous stirring over 12 h. Once cooled, the resulting suspension was centrifuged at 5000 rpm for 5 min (High-Speed Brushless Centrifuge MPW-350, Warsaw, Poland). The separated aqueous extract was concentrated under reduced pressure until slight turbidity appeared, reaching approximately 20% of the original volume. It was then combined with 96% ethanol in a 1:1 volume ratio and allowed to stand overnight at 4 °C. The precipitate was removed by centrifugation (6800 rpm, 20 min), and three volumes of ethanol were added to the supernatant, which was stored overnight at 4 °C. The brown sediment of the initial Se-FE fraction (corresponding to Chihara’s fraction E) was collected by centrifugation, followed by lyophilisation; it was then dissolved in water (20:1 *v*/*w*) and centrifuged again to remove insoluble impurities [17]. Small portions of aqueous CTA-OH were then added until no further sediment formed (pH 12.6). After discarding the sediment, three volumes of ethanol were added to the supernatant, and the mixture was left overnight at 4 °C. Following centrifugation and vacuum drying over P_2_O_5_, the brown sediment was redissolved in deionised water and deproteinated using Sevag reagent (butyl alcohol/chloroform, 1:3 *v*/*v*) [18]. After extensive dialysis using a Spectra/Por^®^ 7 dialysis membrane (MWCO 1000 Da, 45 mm; Carl Roth, Karlsruhe, Germany), the crude Se-FE-1 fraction was obtained by adding three volumes of ethanol, followed by lyophilisation. The reference crude polysaccharide fraction, FE-1, was extracted from *L. edodes* mycelium cultivated in a medium not enriched with Se, using the same method.

#### 2.2.2. Ion-Exchange Chromatography

Fractionation of the crude Se-FE-1 and FE-1 fractions was performed using ion-exchange chromatography on DEAE-Sephadex A-50 resin (GE-Healthcare Bio-Sciences, Uppsala, Sweden). Gradient elution was carried out with 20 mM Tris-HCl buffer (Sigma-Aldrich, St. Louis, MO, USA) with increasing ionic strength, sequentially containing 0.15 M, 1 M, and 2 M NaCl (Sigma-Aldrich, St. Louis, MO, USA). The carbohydrate content in 5 mL fractions collected from the column during chromatographic separation was determined in real time using a previously described densitometric method [19]. Specifically, 3 μL aliquots were taken from each sample using disposable capillary micropipettes and spotted onto TLC plates (Kieselgel 60 F254; Merck Millipore, Darmstadt, Germany). The plates were then visualised by spraying with a reagent composed of 1 g orcinol (Sigma-Aldrich, St. Louis, MO, USA), 50 g absolute alcohol (Chempur, Piekary Śląskie, Poland), and 50 g concentrated sulfuric acid (VI) (Merck Millipore, Darmstadt, Germany), followed by heating for 3 min at 125 °C. Finally, the plates were scanned at a wavelength of 560 nm using a Camag TLC Scanner III (Muttenz, Switzerland). The absorbance of purple-stained spots correlated with the polysaccharide content, enabling the determination of elution profiles for individual polysaccharide subfractions. The groups of eluates corresponding to individual polysaccharides were concentrated under reduced pressure at 50 °C and lyophilised.

#### 2.2.3. Gel-Filtration Desalting Chromatography

**Figure 2:**
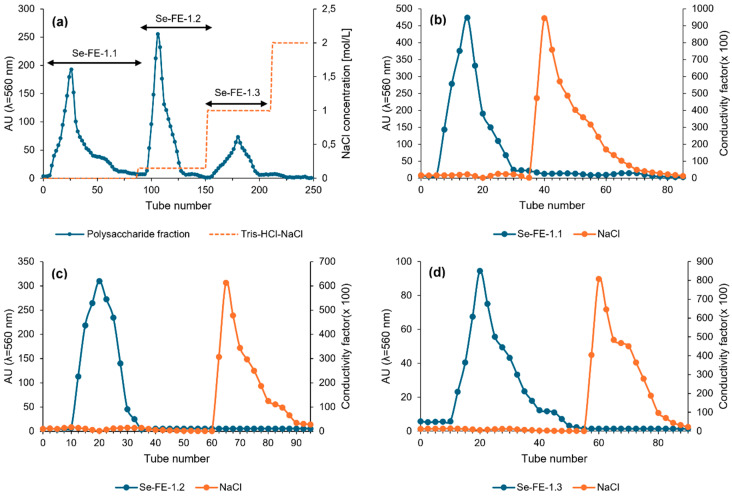
(**a**) Stepwise ion-exchange chromatography profile of the crude Se-FE-1 polysaccharide fraction on DEAE-Sephadex A-50 resin. The three distinct peaks correspond to polysaccharide fractions Se-FE-1.1, Se-FE-1.2, and Se-FE-1.3 eluted with Tris-HCl buffer at increasing ionic strength (0–1 M NaCl). (**b**–**d**) Elution profiles of Se-FE-1.1, Se-FE-1.2, and Se-FE-1.3 during purification on a Sephadex G-25 desalting column.

To remove residual NaCl, the polysaccharide sample solutions were desalinated using a Sephadex G-25 column (50 × 120 mm; Cytiva, Marlborough, MA, USA). Gravity-driven flow with ultrapure water (resistivity 18.2 MΩ·cm) served as the mobile phase, and real-time monitoring was performed using densitometry and conductometry (Figure 2). After combining the eluates, the solution was concentrated via vacuum evaporation at 50 °C and subsequently precipitated with 96% ethanol at 4 °C. The resulting polysaccharides (Se-FE-1.1, Se-FE-1.2, and Se-FE-1.3), along with their non-selenised counterparts (FE-1.1, FE-1.2, and FE-1.3), were then centrifuged, lyophilised, and stored in a desiccator before analysis.

### 2.3. Assessment of Homogeneity and Molecular Weight Measurement

#### High-Performance Gel Permeation Liquid Chromatography (HPGPC) with Evaporative Light-Scattering Detection

The molecular weight and homogeneity of the polysaccharide fractions were determined using HPGPC with evaporative light-scattering detection, following the procedure outlined by Cheong et al. [20], with modifications. The GPC system (Shimadzu, Kyoto, Japan) included two HPLC pumps (Shimadzu, Kyoto, Japan), a GPC column (Ultrahydrogel Linear, 7.8 × 300 mm, Waters, Milford, MA, USA) with an appropriate guard column, a column oven (Shimadzu, Kyoto, Japan), and an evaporative light-scattering detector (ELSD-LTII). Deionised water served as the mobile phase. The column and detector temperatures were set at 70 °C and 80 °C, respectively. The flow rate was 0.5 mL/min, and the injection volume was 20 μL. β-glucan standards (molar masses of 35.6, 70.6, 229, 265, 391, and 650 kDa; Megazyme, Wicklow, Ireland) were used to construct the calibration curve, correlating the retention time with the molecular weight of the standards.

### 2.4. Total Se Analysis in Se–Polysaccharides

#### 2.4.1. Sample Preparation

The total Se determination method followed the protocol outlined by Turło et al. [21]. Each sample (50 mg) underwent triplicate measurements before being transferred to a Teflon crucible. Subsequently, 5 mL of 65% HNO_3_ was added to facilitate mineralisation via microwave digestion. Once digestion was complete, the resulting solution was quantitatively transferred to a 25 mL volumetric flask and diluted with demineralised water. Next, 1 mL of 4 M hydrochloric acid was added to each sample, followed by heating for approximately 30 min at 150 °C to reduce selenite (VI) to selenate (IV) under acidic conditions. After cooling, the contents were transferred to sealed ampoules. The contents of each ampoule were then quantitatively transferred to 20 mL tubes, and 1 mL of 0.09 M EDTA solution was added to chelate any interfering ions. The pH of each sample was adjusted to a range of 1.8–2.1 using 7 M NH_3_ solution with cresol red as an indicator, and samples were diluted to 10 mL with water. Subsequently, 1 mL of DAN reagent (2,3-diaminonaphthalene, 0.1% in 0.1 M HCl solution) was added to each sample, followed by incubation in a water bath at 50 °C for 45 min. After incubation, 3 mL of cyclohexane was added to the samples and shaken for 1 min. The resulting complex was then extracted into the organic layer, collected, and used for Se quantification. A blank sample was prepared using the same procedure, excluding the test sample. Calibration curves were constructed using standard Se solutions (5, 10, 50, 125, 250, 500, and 1000 ng Se/mL) prepared under identical conditions, except for pH adjustments using 0.1 M HCl.

#### 2.4.2. High-Performance Liquid Chromatography with Fluorescence Detection (RP-HPLC/FLD)

RP-HPLC analysis was performed using the Shimadzu LC-10ATvp gradient system, equipped with a fluorescence detector (La Chrom FL-Detector L7480 Merck–Hitachi, Darmstadt, Germany), an SCL-10-Avp system controller, and a CTO-10ACvp column oven. Compounds were separated on a Supelcosil DB (Supelco, Bellefonte, PA, USA) RP-18 column (250 mm × 4.6 mm, 5 μm particle size), with acetonitrile as the mobile phase at a flow rate of 1.4 mL/min. The injection volume was 20 μL, and the analysis was conducted at 25 °C. Fluorometric analysis used an excitation wavelength of 378 nm and an emission wavelength of 557 nm.

### 2.5. Determination of Protein Content

The protein content was assessed spectrophotometrically using the Bradford method [22] on a UV–vis spectrophotometer (UVmini-1240; Shimadzu, Kyoto, Japan), with bovine serum albumin as the standard.

### 2.6. Monosaccharide Composition

#### 2.6.1. Sample Preparation

The monosaccharide composition was determined as previously described [12]. Each polysaccharide sample (25 mg) underwent hydrolysis in sealed glass ampoules with 4 mL of trifluoroacetic acid (TFA) (Sigma-Aldrich Co., St. Louis, MO, USA) at 120 °C for 2 h. After hydrolysis, the samples were cooled, and TFA was removed via evaporation at 60 °C, under vacuum. To ensure complete removal of TFA, water was added and evaporated repeatedly until the pH was neutral (pH 7). The resulting hydrolysates were quantitatively transferred to 10 mL volumetric flasks, brought to volume with HPLC-grade water, and then subjected to derivatisation with 3-methyl-1-phenyl-2-pyrazolin-5-one (PMP; Sigma-Aldrich, St. Louis, MO, USA).

For PMP derivatisation, 120 μL of the sample was mixed with 150 μL of 0.5 M methanolic solution of PMP and 30 μL of 1.5 M NaOH. The mixture was incubated at 70 °C for 2 h in a water bath, shielded from light. After cooling, 100 μL of 0.5 M HCl was added to neutralise the base. The resulting solution was extracted five times with 1 mL of chloroform (ChemPUR GmbH, Karlsruhe, Germany) under intensive stirring to remove excess PMP. The aqueous phase was then analysed by HPLC. The monosaccharide content was determined using standard curves generated from a mixture of D-mannose, D-glucosamine, D-ribose, D-rhamnose, D-glucuronic acid, D-galactosamine, D-glucose, D-galactose, D-xylose, and D-fucose, with standard concentrations ranging from 62.5 to 750 nmol/mL.

#### 2.6.2. HPLC Determination

To determine the monosaccharide composition of polysaccharide samples, an HPLC system (Shimadzu, Kyoto, Japan) was used. The system comprised two LC-10 ATvp pumps, an SCL-10 Avp controller, and an SPD-10 Avp UV detector. A Luna C-18(2) column (5 μm pore size, 250 × 4.6 mm, Phenomenex Inc., Torrance, CA, USA) was employed for the analysis. The samples were eluted at a flow rate of 1.0 mL/min using a mobile phase consisting of 0.1 M phosphate buffer (pH 7.2), buffer A (10% acetonitrile), and buffer B (25% acetonitrile). UV detection was performed at 245 nm. Monosaccharides were separated using a linear gradient of buffer B, starting at 0% at the beginning, increasing to 20% at 10 min, and reaching 100% from 50 to 55 min. The column was maintained at 25 °C, with an injection volume of 20 μL.

### 2.7. IR Spectral Analysis

The FT-IR spectra of dried and ground polysaccharides were recorded using a Perkin Elmer Spectrum Two FT-IR Spectrometer (ATR FT-IR) (Perkin Elmer L160000F; Waltham, MA, USA), equipped with a Universal ATR (attenuated total reflectance) sampling device containing monolithic diamond. The pressure applied to press the powdered sample against the diamond was approximately 100 ± 5 N. Spectra were acquired and processed with Spectrum^TM^ 10 software. The spectra were scanned at room temperature in transmission mode over the frequency range of 4000–400 cm^−1^, with a scan speed of 0.20 cm/s, 15 accumulations at a resolution of 4 cm^−1^, and a data interval of 1 cm^−1^. A background spectrum of air was scanned under the same instrumental conditions prior to measurements.

Additionally, the samples were ground with KBr powder to a thickness of 1 mm for measurement between 4000 and 400 cm^−1^ (scan speed of 0.20 cm/s, resolution of 4 cm^−1^) against pure KBr as a background.

### 2.8. Antioxidant Activity Assay

The antioxidant activity was assessed based on the evaluation of scavenging activity on DPPH free radicals, reductive potential, and the ability to chelate ferric ions. All measurements were performed in triplicate and averaged. The reference antioxidants used for comparison—ascorbic acid, butylated hydroxyanisole (BHA), butylated hydroxytoluene (BHT), α-tocopherol, citric acid, and ethylenediaminetetraacetic acid (EDTA)—were purchased from Sigma-Aldrich (St. Louis, MO, USA).

#### 2.8.1. Determination of DPPH Radical Scavenging Activity

The radical scavenging activity of polysaccharides was evaluated according to the procedure described by Tseng et al. [23], with slight modifications. In total, 200 µL of a methanolic DPPH solution (Sigma-Aldrich, St. Louis, MO, USA) at a concentration of 1 mM was added to 600 µL of each polysaccharide solution in the concentration range of 0.1–2 mg/mL. The absorbance was measured at 517 nm. The sample solution mixed with methanol without DPPH served as a blank. The percentage of free radical scavenging activity was calculated using the following equation:

(1) $$Scavenging\;ability\;\left[\%\right]=\left(1-\frac{A_{1}-A_{2}}{A_{0}}\right)\times 100$$

where *A*_0_ is the absorbance of the control (water instead of sample); *A*_1_ is the absorbance of the sample; and *A*_2_ is the absorbance of the sample under identical conditions as *A*_1_, with methanol instead of DPPH solution.

#### 2.8.2. Determination of Reducing Power

The reducing power of polysaccharides was determined using a modified version of the method by Oyaizu [24]. Specifically, 200 μL of each polysaccharide solution (0.1–2 mg/mL) was combined with 200 μL of phosphate-buffered saline (0.2 M, pH 6.6) and 200 μL of potassium ferricyanide (1%, *w*/*v*; Sigma-Aldrich, St. Louis, MO, USA). This mixture was incubated at 50 °C for 20 min. Following incubation, 200 μL of trichloroacetic acid (10%, *w*/*v*) and 50 μL of ferric chloride (FeCl_3_, 0.1% *w*/*v*) were added. The absorbance was then measured at 700 nm. Ascorbic acid, α-tocopherol, BHA, and BHT served as positive controls. The reducing power was calculated using the following equation:

(2) $$Reducing\;power=A_{1}-A_{2}$$

where *A*_1_ represents the absorbance of the sample; and *A*_2_ denotes the absorbance of the sample measured under identical conditions as *A*_1_, but using distilled water instead of a FeCl_3_ solution.

#### 2.8.3. Determination of Metal Chelating Ability

The Fe^2+^ chelating activity was assessed following the method described by Xie et al. [25]. Briefly, 50 μL of the sample was mixed with 30 μL of FeCl_2_ (0.3 mM) and 120 μL of ferrozine (0.3 mM) solutions. The mixture was vortexed and incubated at 25 °C for 10 min, after which the absorbance at 562 nm was measured. A decrease in absorbance signifies greater chelating power. EDTA and citric acid were used for comparison. The metal chelating activity was calculated using the following equation:

(3) $$Chelating\;activity\;\left[\%\right]=\left(1-\frac{A_{1}-A_{2}}{A_{0}}\right)\times 100$$

where *A*_0_ is the absorbance of the control (water instead of sample); *A*_1_ is the absorbance of the sample; and *A*_2_ is the absorbance of the sample under identical conditions as *A*_1_, with water instead of FeCl_2_.

### 2.9. Statistical Analysis

All results are presented as the mean of at least three replicates ± standard deviation. The data were analysed using one-way analysis of variance, followed by Tukey’s post hoc test (*p* < 0.05), to determine significant differences between groups. Statistical analyses were performed using STATISTICA 13.3 software.

## 3. Results

### 3.1. Polysaccharide Yield and Selenium Distribution

Fractionation of the deproteinised Se-FE-1 fraction on a DEAE-Sephadex A-50 ion-exchange column yielded three polysaccharide fractions, which were further purified on a Sephadex G-25 desalting column (Figure 2). Because of insufficient quantity, the Se-FE-1.3 fraction was excluded from further studies. As demonstrated in Table 1, the yields of the two remaining fractions (Se-FE-1.1 and Se-FE-1.2) were approximately 15 mg/L of the culture medium, which was slightly lower than the yield of the reference non-Se-enriched polysaccharides obtained using the same method. The purified Se-enriched polysaccharides accounted for approximately 14% of the mass of the crude Se-FE-1 fraction; however, the overall extractability of polysaccharides from the mycelium should be considered low.

Comparing the amount of Se accumulated by the mycelium with its content in the native polysaccharide fraction, Se-FE-1, revealed that the transfer of Se to the polysaccharide fractions within the mycelial cells was low, with only a minor quantity of this element incorporated into the polysaccharide molecules. These results also suggest that most of the Se accumulated by the mycelium might have been bound by other mycelial components or deposited in its elemental form (Se^0^) due to the reduction of toxic Na_2_SeO_3_. At the same time, the Se content in the purified polysaccharide fractions was relatively comparable to its content in the crude Se-FE-1 fraction.

### 3.2. Homogeneity and Molecular Weight

**Figure 3:**
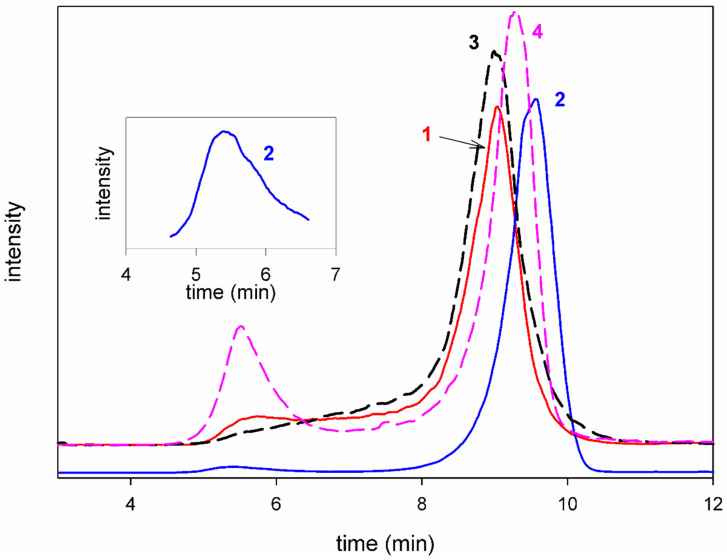
HPGPC chromatograms of the FE and Se-FE fractions. Solid lines represent (1) FE-1.1 and (2) FE-1.2. Dashed lines represent (3) Se-FE-1.1 and (4) Se-FE-1.2. The inset figure shows a fragment of the FE-1.2 chromatogram with increased intensity.

As shown in Figure 3, the HP GPC elution profile of the Se-FE-1.2 polysaccharide displayed a bimodal distribution, with two distinct peaks evident. Additional low-intensity signals (at low retention times) appeared on the chromatograms of the Se-enriched Se-FE-1.1 and the non-Se-enriched fractions. These signals suggested the presence of small quantities of either long polysaccharide chains or aggregates formed from shorter chains. By contrast, the high, narrow, and moderately symmetrical main signals in the upper region of the elution profile indicated a predominant presence of smaller macromolecules with a relatively consistent size distribution.

**Figure 4:**
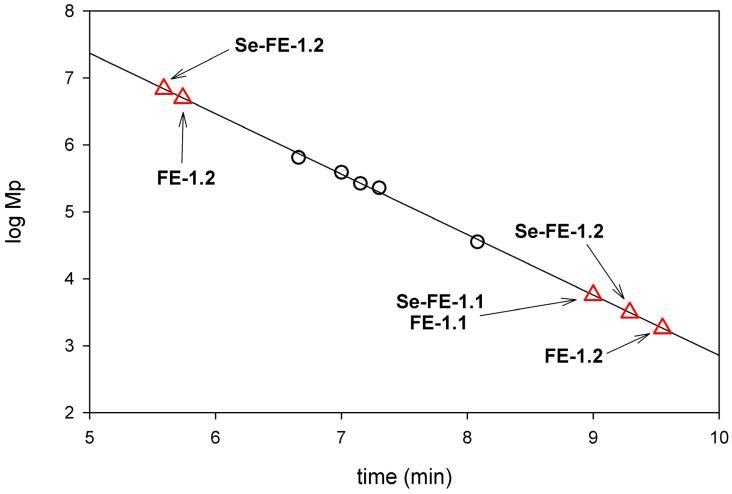
Positions of FE and Se-FE fractions on the calibration line based on β-glucans (HPGPC chromatograms). The symbol ○ denotes β-glucan standards.

The molecular weight of the polysaccharides was determined based on a calibration curve plotted as the log of *Mp* (molecular weight at peak maximum) for individual β-glucan molecular weight standards (Megazyme) against the retention time (Figure 4). The use of β-anomeric polysaccharides was supported by FT-IR analysis results, which showed characteristic bands for β-glucans. The calculated molecular weights for the individual fractions were as follows: FE-1.1 (5.70 × 10^3^ Da), FE-1.2 (1.80 × 10^3^ Da and 5.02 × 10^6^ Da), Se-FE-1.1 (5.70 × 10^3^ Da), and Se-FE-1.2 (3.10 × 10^3^ Da and 6.85 × 10^6^ Da), with the lower values corresponding to the main fractions (high-intensity signal) and the higher values corresponding to the additional fractions (low-intensity signal). However, these values should be interpreted with caution, as they carry uncertainty due to extrapolation, as their elution volume and retention time fell outside the range of standard β-glucans despite the calibration curve demonstrating good linearity and a high coefficient of determination (R^2^ = 0.986).

### 3.3. Chemical Composition

The protein content in the crude Se-FE-1 fraction was 4.70% ± 0.23%. Following additional stages of polysaccharide purification via gel chromatography, the protein content decreased to trace amounts. In the non-Se-enriched fractions, FE-1.1 and FE-1.2, protein was detected at levels of 0.14% ± 0.01% and 0.28% ± 0.03%, respectively, whereas in the selenated fractions, Se-FE-1.1 and Se-FE-1.2, the levels were 0.08% ± 0.01% and 0.37% ± 0.03%, respectively. These results indicate that the purification process effectively removed nearly all free protein.

**Figure 5:**
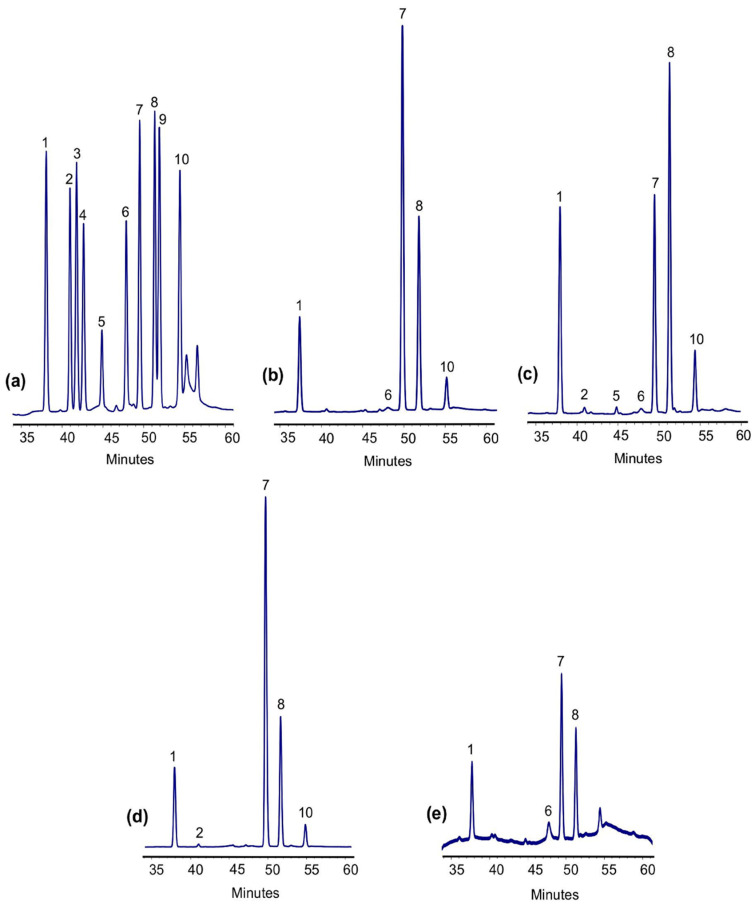
HPLC analysis of the monosaccharide composition of Se-enriched polysaccharides and their non-Se-enriched reference fractions: (**a**) standard sample, (**b**) FE-1.1, (**c**) FE-1.2, (**d**) Se-FE-1.1, and (**e**) Se-FE-1.2. Peaks: (1) mannose, (2) glucosamine, (3) ribose, (4) rhamnose, (5) glucuronic acid, (6) galactosamine, (7) glucose, (8) galactose, (9) xylose, and (10) fucose.

The analysis of the monosaccharide composition of the crude Se-FE-1 fraction revealed a predominance of glucose, mannose, and galactose (in a molar ratio of 1.00:0.37:0.12), with minor amounts of glucosamine, fucose, and xylose (approximately 2.4%, 2.1%, and 0.3%, respectively; Supplementary Materials Figure S1). HPLC chromatograms of monosaccharides in purified Se-FE-1.1 and Se-FE-1.2 polysaccharides, along with their non-Se-enriched counterparts, are shown in Figure 5. Comparative analysis with the chromatogram of a standard monosaccharide mixture indicated that the qualitative composition of all the polysaccharides studied was similar, with prominent peaks corresponding to glucose, galactose, and mannose. These monosaccharides represented around 90–96% of the total polysaccharide mass (Table 2). The molar ratios of glucose, galactose, and mannose were similar in Se-FE-1.1 and FE-1.1 fractions, with values of approximately 5:2:1 and 4:2:1, respectively. However, significant differences were noted between the polysaccharide Se-FE-1.2 and FE-1.2. In the Se-FE-1.2 fraction, glucose was the most abundant monosaccharide, followed by galactose. By contrast, the non-Se-enriched FE-1.2 showed a reversed ratio, with galactose being more prevalent than glucose.

Additionally, all polysaccharides contained amino sugars, although their content was low, with a maximum of approximately 4% in the Se-FE-1.2 fraction. Glucuronic acid was detected in small amounts (approximately 2% by weight) in the FE-1.2 fraction, while trace, negligible amounts were present in the Se-FE-1.1 fraction. All fractions (except Se-FE-1.2) contained the deoxy sugar fucose, with equal amounts in the Se-FE-1.1 fraction and its non-Se-enriched reference (approximately 4% of the total monosaccharide mass).

### 3.4. FTIR Spectra

**Figure 6:**
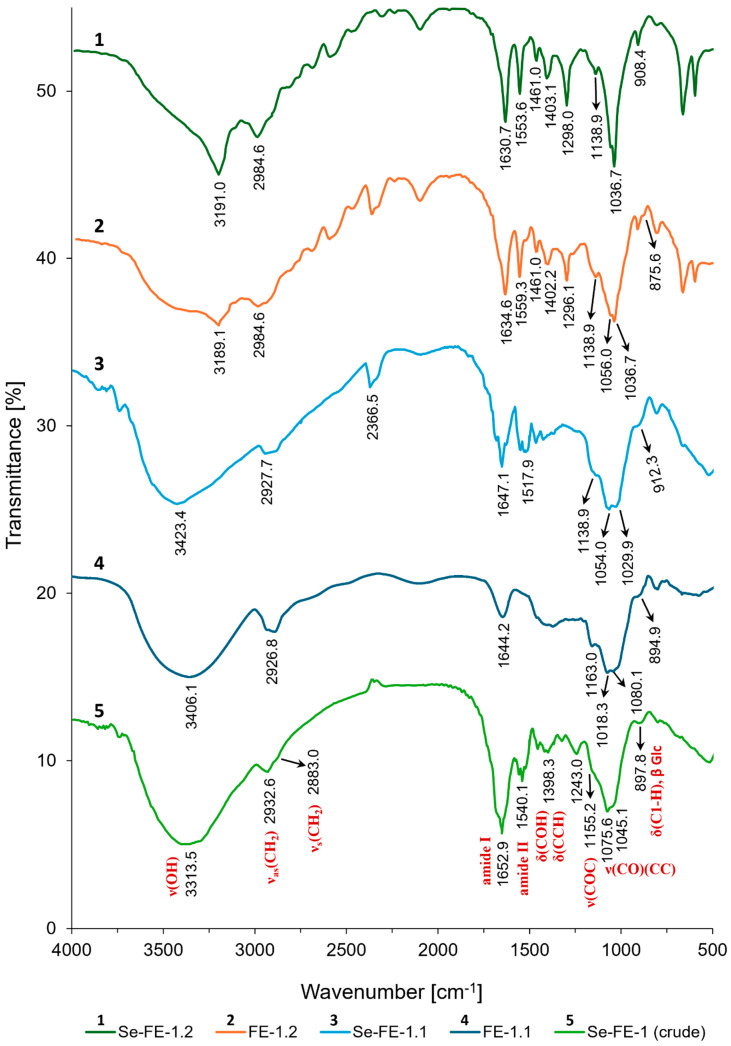
FT-IR spectra of the crude Se-FE-1 polysaccharide fraction; purified Se-enriched polysaccharides, Se-FE-1.1 and Se-FE-1.2; and their non-Se-enriched analogues.

All spectra displayed broad bands between 3191.0 cm^−1^ and 3423.4 cm^−1^, indicative of O–H stretching, which is typical for polysaccharides (Figure 6). These bands suggest extensive hydrogen bonding within the molecules, a characteristic feature of natural polysaccharides. The sharp band at 3191 cm^−1^ in the IR spectra of FE-1.2 and Se-FE-1.2 indicates a reduced number of hydrogen bonds, likely due to the lower molecular weight of the polysaccharides. Smaller polymers with shorter chains have fewer hydroxyl (OH) groups for hydrogen bonding, leading to less extensive intramolecular and intermolecular interactions. Furthermore, the higher proportion of chain ends in smaller polymers increases flexibility and interaction with solvents, reducing the potential for hydrogen bonding within the polymer structure.

The weak peaks at approximately 2360 cm^−1^, as well as the bands around 2928 cm^−1^ and 2985 cm^−1^, were attributed to the symmetric and asymmetric stretching vibrations of skeletal CH and CH_2_ groups in polysaccharides [26,27]. This was consistent across all spectra and indicated the presence of carbohydrate structures.

Bands around 1631–1653 cm^−1^ were assigned to amide I vibrations caused by the presence of N-linked carbonyl groups (C=O) and the in-plane bending of bound water. These bands were common but varied slightly in intensity and shape among the spectra. Additionally, bands near 1540 cm^−1^ in the crude Se-FE-1 fraction, as well as at 1517.9 cm^−1^, 1553.6 cm^−1^, and 1559.3 cm^−1^ in the Se-FE-1.1, Se-FE-1.2, and FE-1.2 fractions, respectively, were characteristic of amide II N–H deformation. These results may indicate the presence of chitin residue, a minor component of the mushroom cell wall, or amino sugars (such as N-acetylglucosamine and galactosamine). Additionally, these bands could suggest protein contamination or the presence of proteins due to the formation of polysaccharide–protein conjugates [27,28,29].

However, it is plausible that the band around 1630 cm^−1^ may also indicate carbonyl groups (C=O) associated with oxidised sugar forms, specifically COO^−^ antisymmetric stretching [4,30]. This interpretation is consistent with the HPLC carbohydrate analysis results, which revealed trace amounts of glucuronic acid in the FE-1.2 and Se-FE-1.1 fractions. Additionally, this could explain the presence of signals around 1400 cm^−1^, corresponding to COO^−^ symmetric stretching.

The band located at 1461–1463 cm^−1^, observed primarily in the FE-1.2 and Se-FE-1.2 fractions, was assigned to the in-plane CH_2_ scissoring motions (bending) in CH_2_OH groups of sugars [27,29,31].

In the literature, researchers primarily attribute the spectral bands in the 1200–1440 cm^−1^ range to in-plane ring deformations involving CH and OH bending modes [27,32]. We observed distinct bands within this range at 1402–1403 cm^−1^ and 1296–1297 cm^−1^ in the FE-1.2 and Se-FE-1.2 fractions. However, because of overlapping bands from various vibrational modes, this spectral region was densely populated, making it challenging to accurately assign the observed bands using conventional group–frequency correlations. Therefore, the prominent band at 1402–1403 cm^−1^ may have also originated from symmetric COO^−^ stretching vibrations in amino acid side chains and carboxylated polysaccharides [33].

In the IV (fingerprint) region, we identified stretching vibrations of C–O–C groups, characteristic of ether-linked glycosidic bonds typical for pyranose rings [26]. Specifically, we observed absorption bands at 1163 cm^−1^, 1080.1 cm^−1^, and 1018.3 cm^−1^ (FE-1.1); 1138.9 cm^−1^, 1056 cm^−1^, and near 1037 cm^−1^ (FE-1.2 and Se-FE-1.2); and 1054.0 cm^−1^, 1138.9 cm^−1^, and 1029.9 cm^−1^ (Se-FE-1.1). The presence of these three absorption peaks, indicative of the pyranose form, is consistent with reports by other authors who have documented similar wavenumber values [9,34,35].

The vibrational bands at 908–913 cm^−1^, described in some of the literature as characteristic of polysaccharides with a beta-anomeric configuration, may have arisen from both asymmetric skeletal vibrations and out-of-plane bending deformations of the C1–H bond in polysaccharides [36,37,38]. However, definitive confirmation of the beta configuration was possible in the case of the selenated crude Se-FE-1 fraction, as well as the non-selenated FE-1.1 and FE-1.2 fractions, where FT-IR analysis revealed low-intensity shoulders at 897.8 cm^−1^, 894.9 cm^−1^, and 875.6 cm^−1^, respectively, typical of the beta-anomer in glucopyranoses [9,26,27,35,39,40,41]. The presence of beta-glycosidic bonds was further confirmed by comparing the polysaccharide spectra with the FT-IR spectrum of a (1,3)(1,4)-β-glucan molecular-weight standard (Megazyme, CAS Number 9041-22-9), which exhibited a characteristic peak at 896.1 cm^−1^ in the anomeric region (Supplementary Materials Figure S2). Notably, based on multiple analyses of the Se-FE-1 fraction spectrum, we also observed that the anomeric band at 897.8 cm^−1^ could shift slightly toward higher wavenumber values (Supplementary Materials Figure S3). This may confirm that the peak observed in the Se-FE-1.1 and Se-FE-1.2 fractions around 908–913 cm^−1^ indicated the presence of beta-glycosidic bonds.

### 3.5. Antioxidant Activity

**Figure 7:**
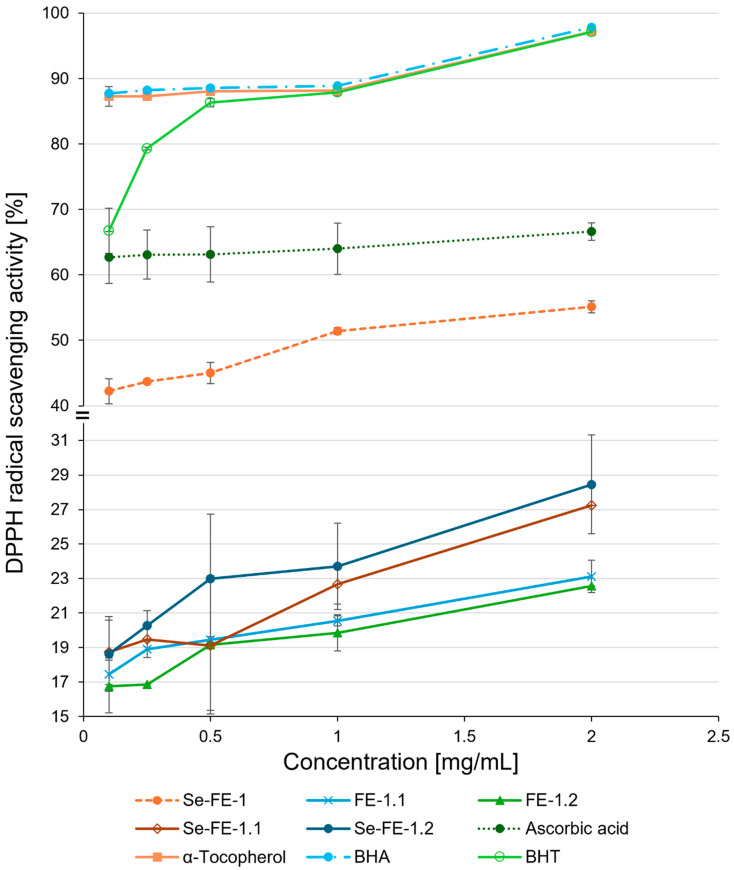
DPPH radical scavenging ability of purified Se-enriched polysaccharides, Se-FE-1.1 and Se-FE-1.2, compared with their unmodified non-Se-enriched analogues, FE-1.1 and FE-1.2; the crude Se-FE-1 fraction; and reference antioxidants. Each value represents the mean ± standard deviation (n = 3).

The ability of polysaccharides to scavenge the DPPH free radical increased gradually with rising concentrations (Figure 7). At a concentration of 2 mg/mL, the scavenging activity of the Se-enriched polysaccharides Se-FE-1.1 and Se-FE-1.2 was 27.25% and 28.45%, respectively, which was slightly but statistically significantly higher than that of the non-Se-enriched polysaccharides (23.12% for FE-1.1 and 22.57% for FE-1.2). All tested reference antioxidants exhibited substantially higher activity, with BHA showing the highest activity, approximately 3.5 times greater than the Se-enriched polysaccharides.

**Figure 8:**
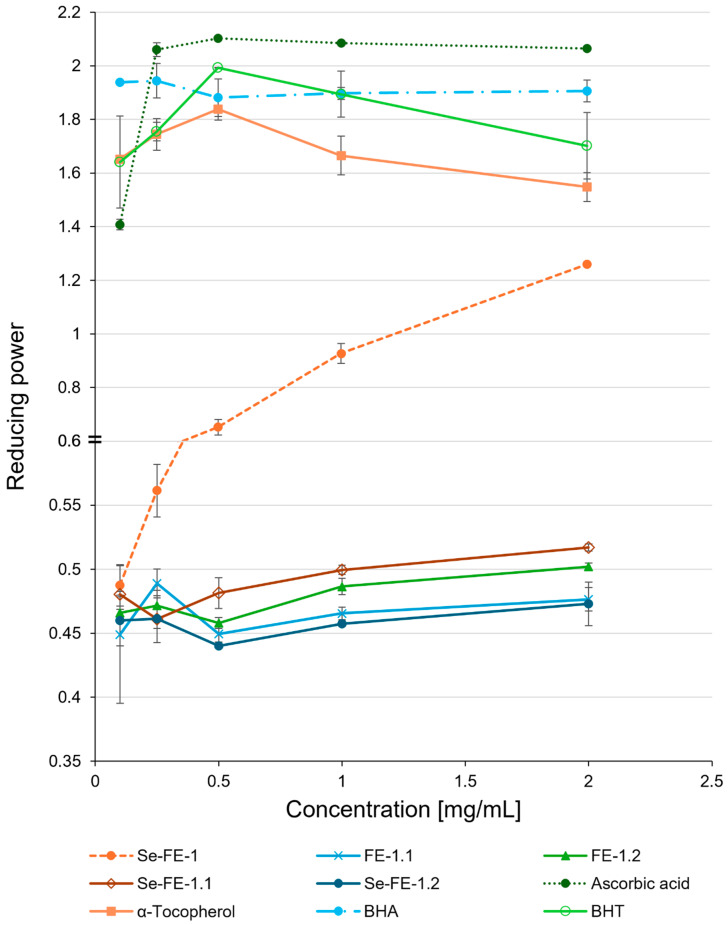
Reducing power of purified Se-enriched polysaccharide fractions, Se-FE-1.1 and Se-FE-1.2, compared with their unmodified non-Se-enriched analogues, FE-1.1 and FE-1.2; the crude Se-FE-1 fraction; and reference antioxidants. Each value represents the mean ± standard deviation (n = 3).

The purified Se-enriched polysaccharides demonstrated low reducing power across all concentrations, comparable to their non-selenated counterparts (Figure 8). Among all the reference antioxidants, ascorbic acid proved to be the most effective, with a reducing power of 2.07 at the highest tested concentration (2 mg/mL), which was, on average, four times greater than that of all purified polysaccharides. Interestingly, the reducing power of the crude Se-FE-1 fraction increased with concentration, ultimately reaching a value of approximately 1.26, about 39% lower than ascorbic acid and only 19% lower than α-tocopherol. This value was also more than 2.5 times higher than the average activity of the purified polysaccharides.

**Figure 9:**
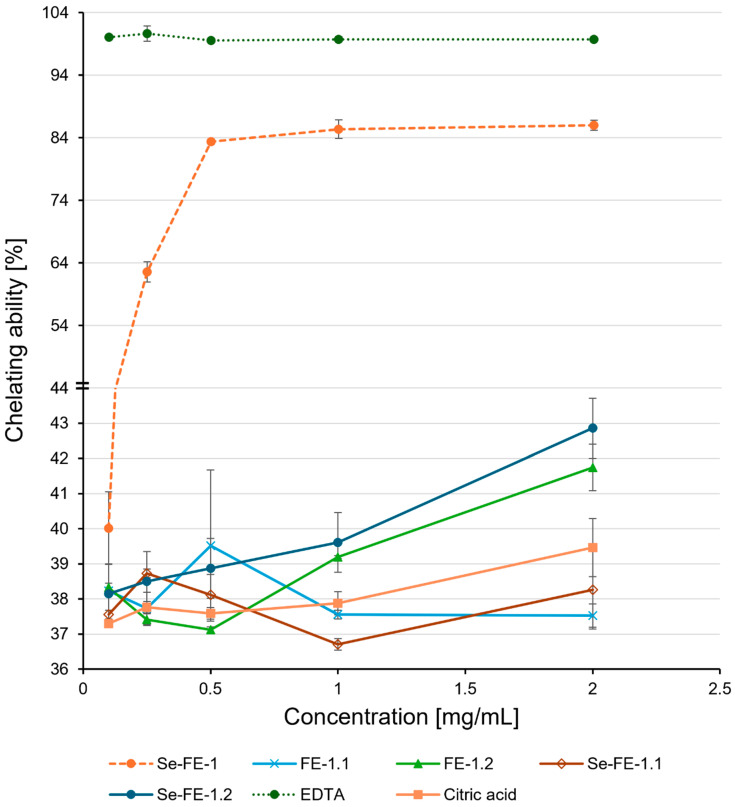
Fe^2+^ chelating ability of purified Se-enriched polysaccharide fractions, Se-FE-1.1 and Se-FE-1.2, compared with their unmodified non-Se-enriched analogues, FE-1.1 and FE-1.2; the crude Se-FE-1 fraction; and reference antioxidants. Each value represents the mean ± standard deviation (n = 3).

The study also showed similar results regarding ferrous ion chelating ability (Figure 9). At the highest concentration, the crude Se-FE-1 fraction exhibited approximately twice the Fe^2+^ binding capacity compared with the purified polysaccharides. Initially, the chelation ability of the crude Se-FE-1 fraction increased with concentration, but at 0.5 mg/mL, it reached a stable level (approximately 85%), about 15% lower than that of EDTA, which was used as a reference. The chelating ability of the purified polysaccharide fractions remained constant at approximately 40%, comparable to that of citric acid, with peak activity observed at the lowest concentration tested (0.1 mg/mL). The results did not reveal significant differences between the Se-enriched and non-Se-enriched polysaccharides across the tested concentration range. Although the chelating abilities of all purified polysaccharides appeared similar, statistically significant differences (*p* ≤ 0.05) indicated slightly higher activity in the FE-1.2 and Se-FE-1.2 fractions than in both the Se-FE-1.1 polysaccharide and its unmodified FE-1.1 counterpart.

## 4. Discussion

Building on our previous research, we hypothesised that incorporating Se into the polysaccharide structure would enhance antioxidant effects, as observed for a Se-enriched lentinan analogue [16]. Based on these findings, we expected similar results for other Se–polysaccharides biosynthesised using *L. edodes* mycelium grown on a Se-enriched medium. Our supposition also aligns with findings from other researchers, who demonstrated that polysaccharides containing Se have more pronounced antioxidant activity [13]. Incorporating Se activates hydrogen atoms and alters the molecular weight, spatial structure, and physicochemical properties, thereby influencing biological activity [42]. However, our results deviated from expectations, as the antioxidant activity depends on multiple factors, including structural and chemical features [39], which may have contributed to the observed differences, rather than Se incorporation alone. Therefore, alongside investigating the effect of Se incorporation, we examined the structural characteristics of both Se-enriched and non-Se-enriched polysaccharides and explored their relationship with antioxidant activity.

As shown in Table 1, the Se content in the crude Se-FE-1 polysaccharide fraction was approximately 84 µg/g and remained relatively stable after purification and fractionation. In the purified Se-FE-1.1 and Se-FE-1.2 polysaccharides, the Se concentrations were around 125 µg/g and 82 µg/g, respectively. This finding suggests that Se is covalently bonded within the polysaccharide structure rather than being superficially adsorbed on its surface or forming transient complexes with the polysaccharide chains. The similar Se content in both Se-enriched polysaccharide fractions (with a slight advantage for Se-FE-1.1) suggests a relatively uniform distribution of Se within the initial crude Se-FE-1 fraction. Given that the initial Se-FE-1 fraction contains only a minor amount of protein (approximately 5%), we concluded that nearly all of the selenium is likely incorporated into the carbohydrate moiety.

Although the Se content was within the upper range reported for natural Se-containing polysaccharides identified as potential antioxidants [11,43,44], the results regarding antioxidant activity differed from our expectations. Among all the isolated compounds, the crude Se-FE-1 polysaccharide exhibited the most pronounced antioxidant activity across all three in vitro assays (DPPH radical scavenging, reducing power, and ferrous ion chelation). The Se-enriched polysaccharides obtained through subsequent fractionation exhibited significantly weaker activity, with their reducing potential and ferrous ion chelation abilities lower than those of the crude Se-FE-1 fraction and comparable to the non-Se analogues (Figure 7). The Se-enriched polysaccharides were more effective than their non-Se counterparts only in the DPPH radical scavenging test, achieving moderate values (up to approximately 28.5% for the Se-FE-1.2 fraction), which are lower than those of well-known antioxidants. Compounds with higher DPPH radical scavenging rates donate hydrogen atoms, forming stable DPPH-H molecules. Polysaccharides transfer hydrogen atoms to reduce DPPH radicals, and the reduction extent correlates with the number of available OH groups. Our results show that Se-enriched polysaccharides (Se-FE-1.1 and Se-FE-1.2) donate more OH groups than their non-Se counterparts (FE-1.1 and FE-1.2), exhibiting greater radical scavenging capacity, likely due to their distinct structure.

Beyond their ability to scavenge free radicals, antioxidants also demonstrate reducing power and chelating ability, which enhance their effectiveness in mitigating oxidative damage. Reducing power refers to the capacity of a substance to donate electrons and neutralise free radicals directly. By contrast, chelating involves binding metal ions, such as ferrous ions, to prevent their involvement in reactions that generate free radicals. Both mechanisms contribute to reducing oxidative stress by either directly neutralising reactive species or controlling the metal ions that catalyse their formation [45]. The lack of significant differences in reducing power and chelating ability between the Se-enriched fractions Se-FE-1.1 and Se-FE-1.2 and their non-Se counterparts suggests that the Se content does not affect the antioxidant activity of these polysaccharides. Considering the discrepancies between our current findings and previous studies, the antioxidant activity of Se–polysaccharides may depend on how Se is bound to the molecule. For instance, Wei et al. [46] demonstrated that the higher antioxidant activity of synthetic polysaccharides with C-6 ester-linked selenious acid results from the oxidation state of Se changing from Se^4+^ to Se^6+^. Our earlier studies on other biosynthesised selenised polysaccharides showed that Se is likely incorporated in the –II oxidation state and bound through β-1,3- or α-1,4-glycosidic bonds or may substitute for an oxygen atom in the carbohydrate ring, potentially perturbing the structure of adjacent residues and influencing biological activity [14]. The Se-enriched polysaccharides in this study might have a different mode of Se incorporation, potentially altering their biological activity. Nevertheless, evidence for such a structure in the Se-enriched polysaccharides obtained in this study and its relationship to antioxidant activity still needs to be demonstrated.

Infrared spectra and HPLC analysis of monosaccharide composition revealed that the obtained compounds are heteropolysaccharides mainly composed of glucose, galactose, and mannose, linked by β-glycosidic bonds (Figure 5 and Figure 6; Table 2). The absence of α-glycosidic bonds and the corresponding FT-IR absorption bands in both Se-enriched and non-Se-enriched polysaccharides suggest that Se does not affect the orientation of the OH group at the anomeric carbon in cyclic sugars. Significant differences in the monosaccharide composition between Se-enriched and non-enriched polysaccharides, particularly the shift in the glucose-to-galactose ratio from 0.69:1.00 in the non-enriched FE-1.2 fraction to 1.00:0.73 in the Se-enriched fraction, indicate that Se incorporation may influence the sugar composition. The underlying mechanism requires further investigation.

The type of monosaccharide units is a crucial factor influencing the antioxidant activity of polysaccharides. The obtained polysaccharides had a relatively high glucose-to-mannose ratio, which may have contributed to their antioxidant activity being lower than we expected. Meng et al. [47] reported that while galactose content does not correlate with antioxidant properties, mannose enhances antioxidant activity, and glucose has the opposite effect. In line with this, the fractions with a more balanced mannose-to-glucose ratio (FE-1.2 and Se-FE-1.2) exhibited higher chelating ability, suggesting that monosaccharide composition could influence antioxidant potential. The enhanced chelating ability of the FE-1.2 and Se-FE-1.2 fractions (Figure 9), which had a more balanced mannose-to-glucose ratio compared to the FE-1.1 and Se-FE-1.1 fractions (Table 2), further supports this observation.

The molecular weight plays a key role in antioxidant activity by affecting the availability of functional groups and conformational stability [45]. HPGPC analysis showed that the molecular weights of the examined polysaccharides fell outside the calibration curve (Figure 4), likely due to structural and hydrodynamic differences from the standard beta-glucans. Given the need for extrapolation, these values require cautious interpretation. Despite this limitation, fractions FE-1.1 and Se-FE-1.1 likely contained short chains of several dozen monosaccharide units, whereas Se-FE-1.2 and FE-1.2 appeared to consist of low-molecular-weight polysaccharides or oligosaccharides, with a minor presence of high-molecular-weight components. These molecular weight characteristics may have influenced the antioxidant properties of the obtained polysaccharides. While lower molecular weights are generally associated with enhanced antioxidant activity due to the higher availability of terminal OH groups [48], excessively low molecular weights can compromise structural stability, limiting effectiveness [49]. Our results suggest that the Se-enriched polysaccharides, primarily composed of short chains with some high-molecular-weight aggregates, may not have attained the optimal conformation or functional group accessibility essential for improved antioxidant performance.

The significantly higher antioxidant activity observed in the crude Se-FE-1 fraction, compared to the purified polysaccharides (2–2.5 times greater at the highest concentration), suggests a potential contribution from a protein component. This finding supports research highlighting the role of protein or peptide moieties in enhancing antioxidant activity, as seen in β-glucans from fungi and yeast [50,51]. Some authors emphasise the need to reassess polysaccharides as antioxidants, considering the importance of effective extraction and purification to eliminate the influence of other compounds on their antioxidant properties [48,52]. Our findings support this viewpoint, aligning with their conclusions.

In summary, although previous studies led us to expect that the newly obtained Se-enriched polysaccharides would show higher activity than their non-selenised counterparts across all in vitro antioxidant tests, we observed this enhanced effect only in the DPPH radical scavenging assay. To clarify these discrepancies, we compared our results with previous studies, which allowed us to identify potential reasons:

- The newly biosynthesised Se-enriched polysaccharides, possessing lower molar masses than those described in previous studies [15,16], likely exhibit distinct structural characteristics. Variations in incorporating Se into the polysaccharides may influence their conformation and bioavailability, consequently affecting their ability to neutralise reactive oxygen species. It is conceivable that selenium modifies the structures of polysaccharides Se-FE-1.1 and Se-FE-1.2, possibly by introducing novel functional groups or altering existing ones. As a result, this may enhance their electron-donating capabilities and interactions with DPPH radicals compared to non-Se-enriched analogues. For mechanisms related to metal ion chelation or reduction, the effectiveness may hinge on specific chemical interactions not significantly altered by Se, which may explain the absence of notable differences in these assays.
- In our earlier research, we synthesised a Se-enriched lentinan analogue demonstrating antioxidant activity in HeLa cells. However, in investigations assessing the impact of this compound on superoxide anion (O_2_^−^) production in human peripheral blood granulocytes, we did not observe a significant influence on reactive oxygen species generation in these cells. This observation led us to conclude that this polysaccharide does not affect reactive oxygen species (ROS) production, suggesting that its antioxidant action does not rely on inhibiting ROS generation [15,16]. The results align with the findings described in the current study, where the pronounced activity of selenised polysaccharides Se-FE-1.1 and Se-FE-1.2 was exclusively evident in radical scavenging assays, with no significant activity observed in chelation or reduction tests. This pattern indicates that these polysaccharides act more directly as ‘scavengers’ of radicals, effectively neutralising existing free radicals rather than preventing their generation.
- The presence of non-carbohydrate substances, such as proteins, may significantly influence the biological activity of polysaccharides, including their antioxidant capacity. Such interactions could account for the distinct advantage of the crude Se-FE-1 fraction over purified polysaccharides in all antioxidant activity assays. It also provides insight into the disparity observed between the activities of the newly synthesised Se–polysaccharides, largely devoid of protein, and the previously reported exceptional antioxidant properties of aqueous extracts derived from selenised *L. edodes* mycelium [53].

Overall, the observed differences in antioxidant activity among the selenised polysaccharides isolated from identical submerged cultures of the same strain of fungus *L. edodes* may result from a complex interplay between their chemical structures, Se forms and mechanisms of action. Future studies may provide deeper insights into these mechanisms and enhance our understanding of the role of Se insertion in the structure and function of polysaccharides.

## 5. Conclusions

Selenium can be effectively incorporated into polysaccharides through mycelial biosynthesis in a Se-supplemented medium, demonstrating its potential for obtaining Se-enriched biopolymers.

Our results reveal a complex interplay between selenium incorporation, monosaccharide composition of polysaccharides, and their antioxidant activity. Se-enriched and non-Se polysaccharides showed differences in the weight percentages of their main monosaccharides, suggesting that Se incorporation alters sugar composition, which may influence their antioxidant activity, as seen in the varying chelating abilities of fractions with different mannose-to-glucose ratios. Additionally, preserving glycosidic linkages in Se-enriched polysaccharides indicates that Se did not affect the anomeric arrangement.

Selenium incorporation into polysaccharides contributed to the selective enhancement of their antioxidant activity in the DPPH radical scavenging assay, while its impact on iron chelation and reducing power assays was limited. This outcome suggests that selenium mainly influences radical scavenging mechanisms rather than metal ion interactions.

Given the higher antioxidant activity observed in the crude, unpurified Se-enriched polysaccharide fraction, protein components could play a role in the bioactivity. While optimizing purification processes to isolate the polysaccharides is crucial, further investigation into the contribution of protein interactions to their antioxidant potential is also essential. These insights could aid in refining purification methods that either retain or selectively remove protein components, ensuring a more precise evaluation of Se-enriched polysaccharides for potential applications in functional foods and dietary supplements. Additional research also requires understanding the structural and chemical mechanisms governing Se incorporation into polysaccharides and how these affect their antioxidant properties.

## Figures and Tables

**Table 1:** Polysaccharide extraction yield, selenium content, and Se distribution in *Leninula edodes* Se-enriched polysaccharides.

Sample	Yield (g/10 L) ^a^	Average Extractability (%) ^b^	Se Content (mg/g D.W. *)	Se Content Ratio (%) ^c^
Se–mycelium	109 ± 11	−	1567 ± 17	100
Se-FE-1 (crude)	2.18 ± 0.35	2.00	83.5 ± 3.6	0.106
Se-FE-1.1	0.15 ± 0.02	0.14	124.6 ± 1.4	0.011
Se-FE-1.2	0.153 ± 0.008	0.14	81.7 ± 3.1	0.007
Mycelium	93.0 ± 3.2	−	− ^d^	−
FE-1	1.3 ± 0.2	1.35	− ^d^	−
FE-1.1	0.181 ± 0.016	0.20	− ^d^	−
FE-1.2	0.222 ± 0.019	0.24	− ^d^	−

^*^ Dry weight. ^a^ Yield represents the dry weight of the sample obtained from 10 L of mycelial culture. ^b^ Amount of sample extracted from 100 g of dry mycelial biomass. ^c^ Percentage of Se in the sample relative to the total Se content in the mycelium, calculated as follows: % = (Se content in the sample × sample weight)/(Se content in the mycelium × mycelium dry weight). ^d^ Negligible Se content (<0.1 μg/g).

**Table 2:** Monosaccharide composition of Se-containing polysaccharides (Se-FE-1.1 and Se-FE-1.2) and their non-Se-enriched reference fractions.

Monosaccharide	Percentage *w*/*w*				Molar Ratio **			
	FE-1.1	Se-FE-1.1	FE-1.2	Se-FE-1.2	FE-1.1	Se-FE-1.1	FE-1.2	Se-FE-1.2
D-mannose	13.7 ± 1.2 ^a^ *	13.69 ± 0.91 ^a^	26.6 ± 0.4 ^b^	22.4 ± 0.3 ^c^	0.24	0.22	0.71	0.53
D-glucosamine	n.s.	0.57 ± 0.03 ^a^	0.63 ± 0.04 ^a^	n.d.	−	0.01	0.01	−
D-ribose	n.d.	n.d.	n.d.	n.d.	−	−	−	−
D-rhamnose	n.d.	n.d.	n.d.	n.d.	−	−	−	−
D-glucuronic acid	n.d.	n.s.	2.01 ± 0.14	n.d.	−	−	0.05	−
D-galactosamine	1.14 ± 0.06 ^a^	n.d.	0.56 ± 0.03 ^a^	3.7 ± 1.6 ^a^	0.01	−	0.01	0.07
D-glucose	56.59 ± 0.45 ^a^	62.2 ± 0.6 ^b^	25.80 ± 0.11 ^c^	42.6 ± 0.8 ^d^	1.00	1.00	0.69	1.00
D-galactose	25.3 ± 0.7 ^a^	19.5 ± 0.3 ^b^	37.20 ± 0.23 ^c^	31.23 ± 0.54 ^d^	0.45	0.31	1.00	0.73
D-xylose	n.d.	n.d.	0.08 ± 0.03	n.d.	−	−	−	−
D-fucose	3.75 ± 0.12 ^a^	3.97 ± 0.01 ^a^	7.1 ± 0.2 ^b^	n.d.	0.07	0.07	0.21	−

Data are presented as mean ± standard deviation (n = 3). * Mean values within the same row labelled with different lowercase letters differ significantly (*p* ≤ 0.05, analysis of variance, Tukey’s honestly significant difference test). ** Molar ratio relative to the dominant monosaccharide. n.d.—not detected. n.s.—not significant (<0.1%).

## Data Availability

The raw data supporting the conclusions of this article will be made available by the authors on request.

## Supplementary Materials

The following supporting information can be downloaded at https://www.mdpi.com/article/10.3390/polym17060719/s1, Figure S1: HPLC analysis of monosaccharide composition of the crude Se-FE-1 polysaccharide fraction. (a) Standard sample, (b) crude Se-FE-1. Peaks: (1) mannose, (2) glucosamine, (3) ribose, (4) rhamnose, (5) glucuronic acid, (6) galactosamine, (7) glucose, (8) galactose, (9) xylose, (10) fucose; Figure S2. FT-IR spectrum of (1,3)(1,4)-β-glucan molecular weight standard, showing a characteristic absorption peak confirming the presence of the β-configuration; Figure S3. Variations in the wavenumber of the C1-H bond of the beta-anomer observed in individual IR spectra of the same crude Se-FE-1 polysaccharide sample.
