# Supplementary material for: Selenium-Enriched Polysaccharides from Lentinula edodes Mycelium: Biosynthesis, Chemical Characterisation, and Assessment of Antioxidant Properties
Source: Polymers (Basel). 2025 Mar 9;17(6):719. doi: 10.3390/polym17060719 (PMC11944456; doi:10.3390/polym17060719)
Supplement: Supplementary file 1 [file polymers-17-00719-s001.zip › supplementary material.pdf]

SUPPLEMENTARY MATERIAL

Selenium-Enriched Polysaccharides from *Lentinula edodes* Mycelium: Biosynthesis, Chemical Characterisation, and Assessment of Antioxidant Properties

Eliza Malinowska [1,*], Grzegorz Łapienis [2], Agnieszka Szczepańska [1] and Jadwiga Turło [1]

[1] Department of Drug Technology and Pharmaceutical Biotechnology, Medical University of Warsaw, 1 Banacha Str., 02-097 Warszawa, Poland; agnieszka.szczepanska@wum.edu.pl (A.S.); jadwiga.turlo@wum.edu.pl (J.T.)
[2] Department of Functional Polymers and Polymeric Materials, Centre of Molecular and Macromolecular Studies, Polish Academy of Sciences, 112 Sienkiewicza Str., 90-363 Łódź, Poland; grzegorz.lapienis@cbmm.lodz.pl
* Correspondence: eliza.malinowska@wum.edu.pl; Tel.: +48-225720647

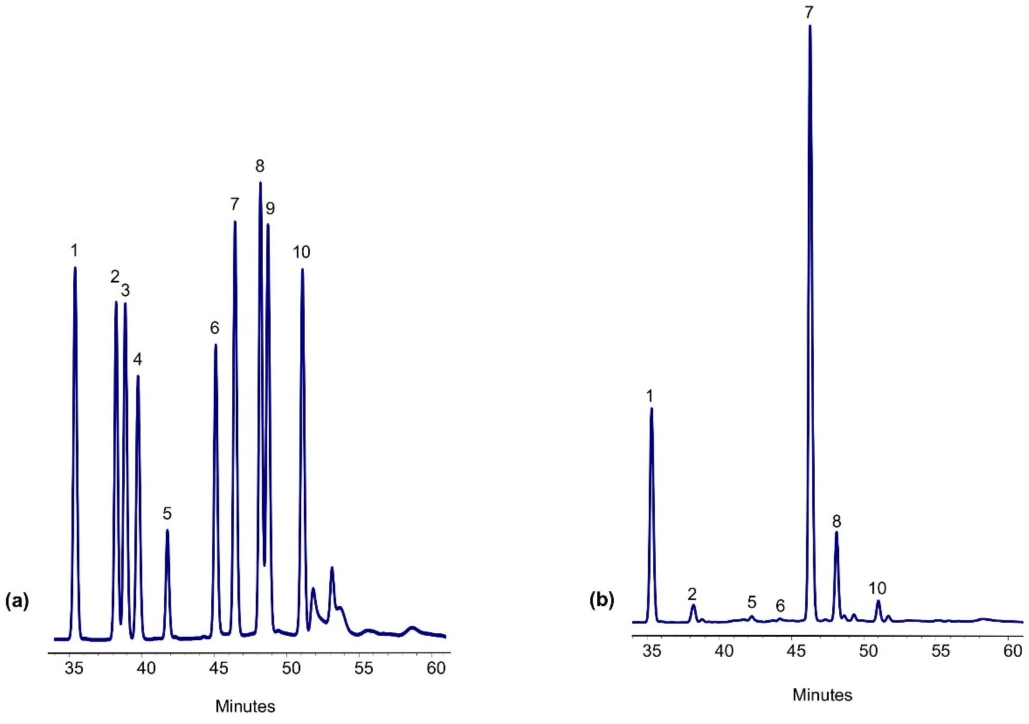

Figure S1. HPLC analysis of monosaccharide composition of the crude Se-FE-1 polysaccharide fraction. (a) Standard sample, (b) crude Se-FE-1. Peaks: (1) mannose, (2) glucosamine, (3) ribose, (4) rhamnose, (5) glucuronic acid, (6) galactosamine, (7) glucose, (8) galactose, (9) xylose, (10) fucose.

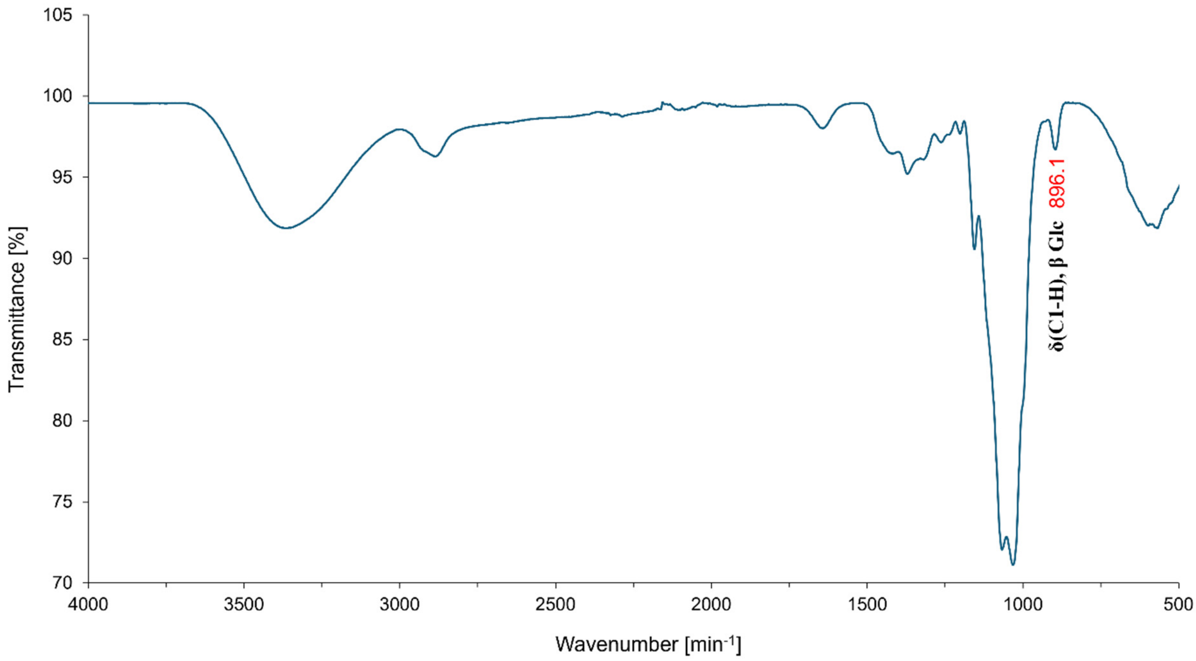

Figure S2. FT-IR spectrum of (1,3)(1,4)-β-glucan molecular weight standard, showing a characteristic absorption peak confirming the presence of the β-configuration.

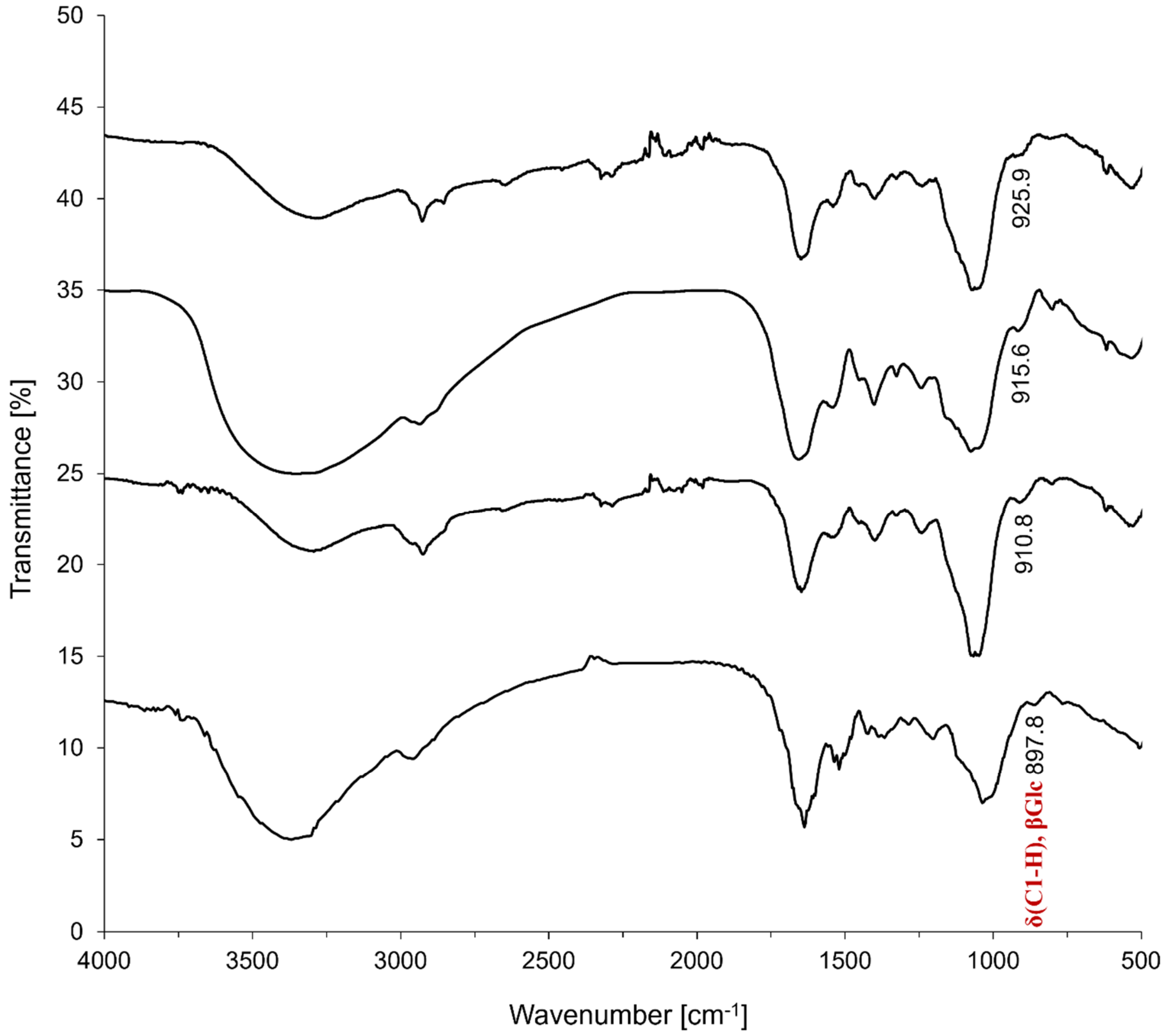

Figure S3. Variations in the wavenumber of the C1-H bond of the beta-anomer observed in individual IR spectra of the same crude Se-FE-1 polysaccharide sample.
