# Supplementary figures and images for: Selenium-Enriched Polysaccharides from Lentinula edodes Mycelium: Biosynthesis, Chemical Characterisation, and Assessment of Antioxidant Properties
Source: Polymers (Basel). 2025 Mar 9;17(6):719. doi: 10.3390/polym17060719 (PMC11944456; doi:10.3390/polym17060719)

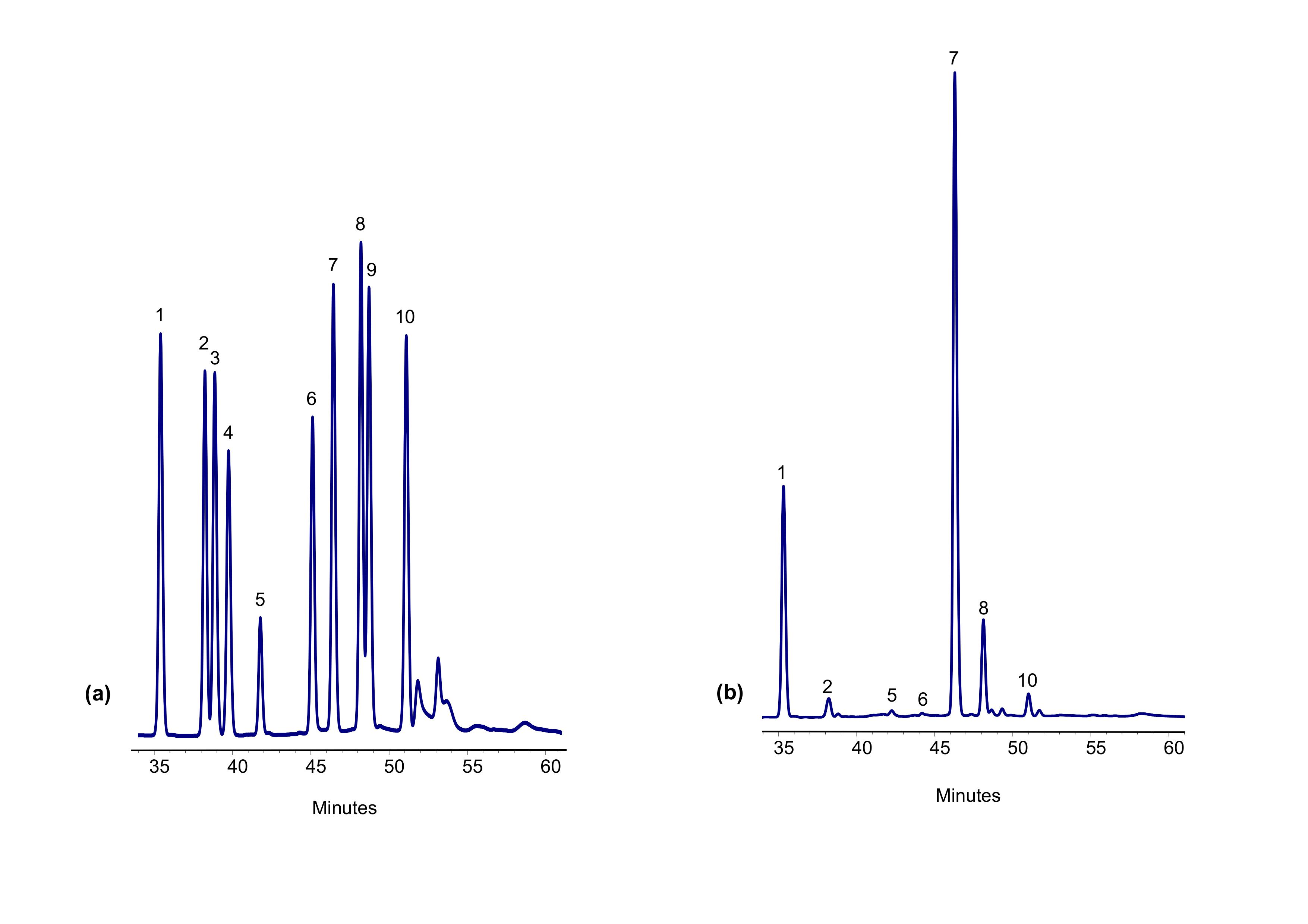

Supplement: Supplementary file 1 [file polymers-17-00719-s001.zip › Figure S1.tiff]

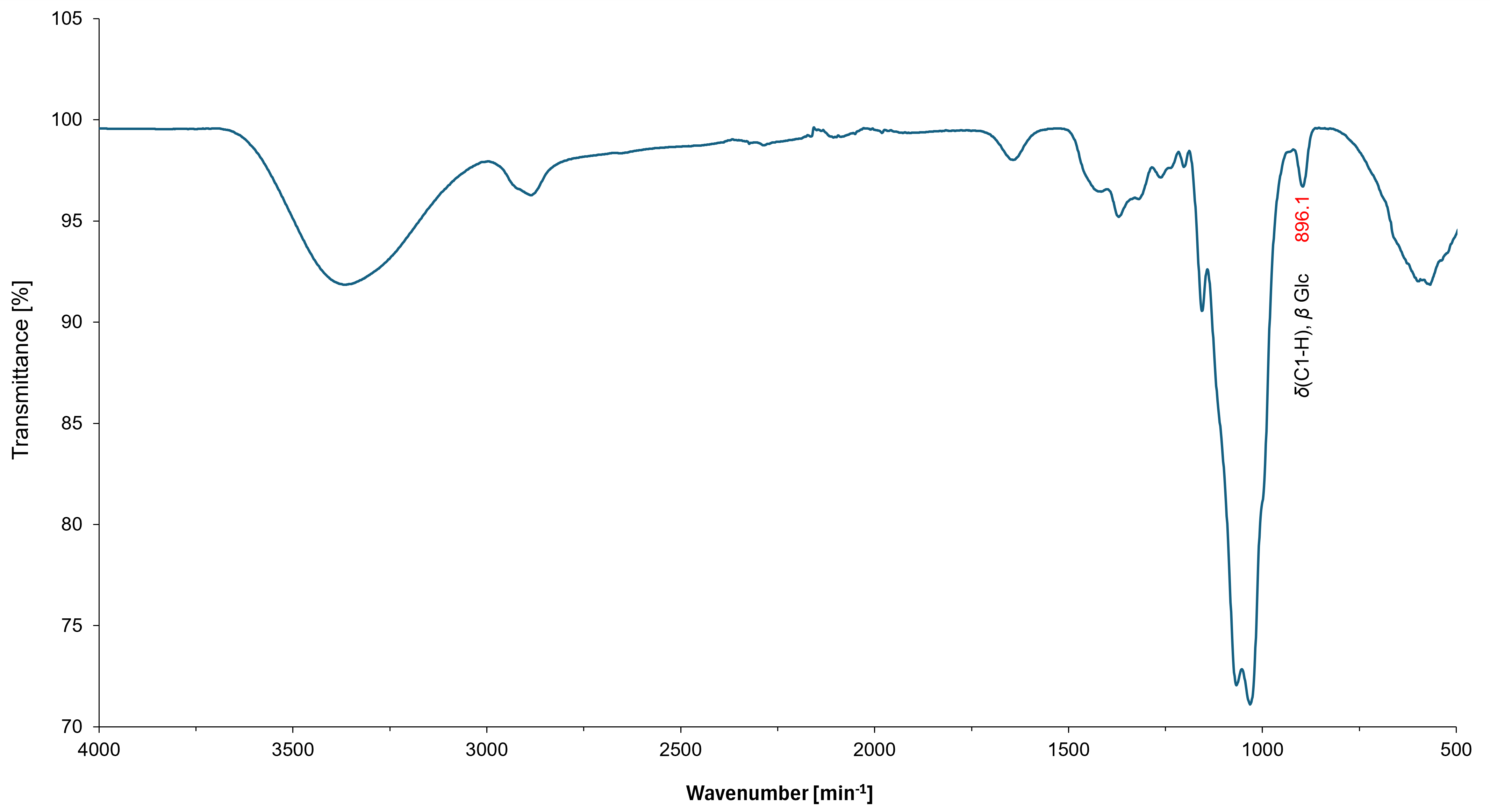

Supplement: Supplementary file 1 [file polymers-17-00719-s001.zip › Figure S2.tif]

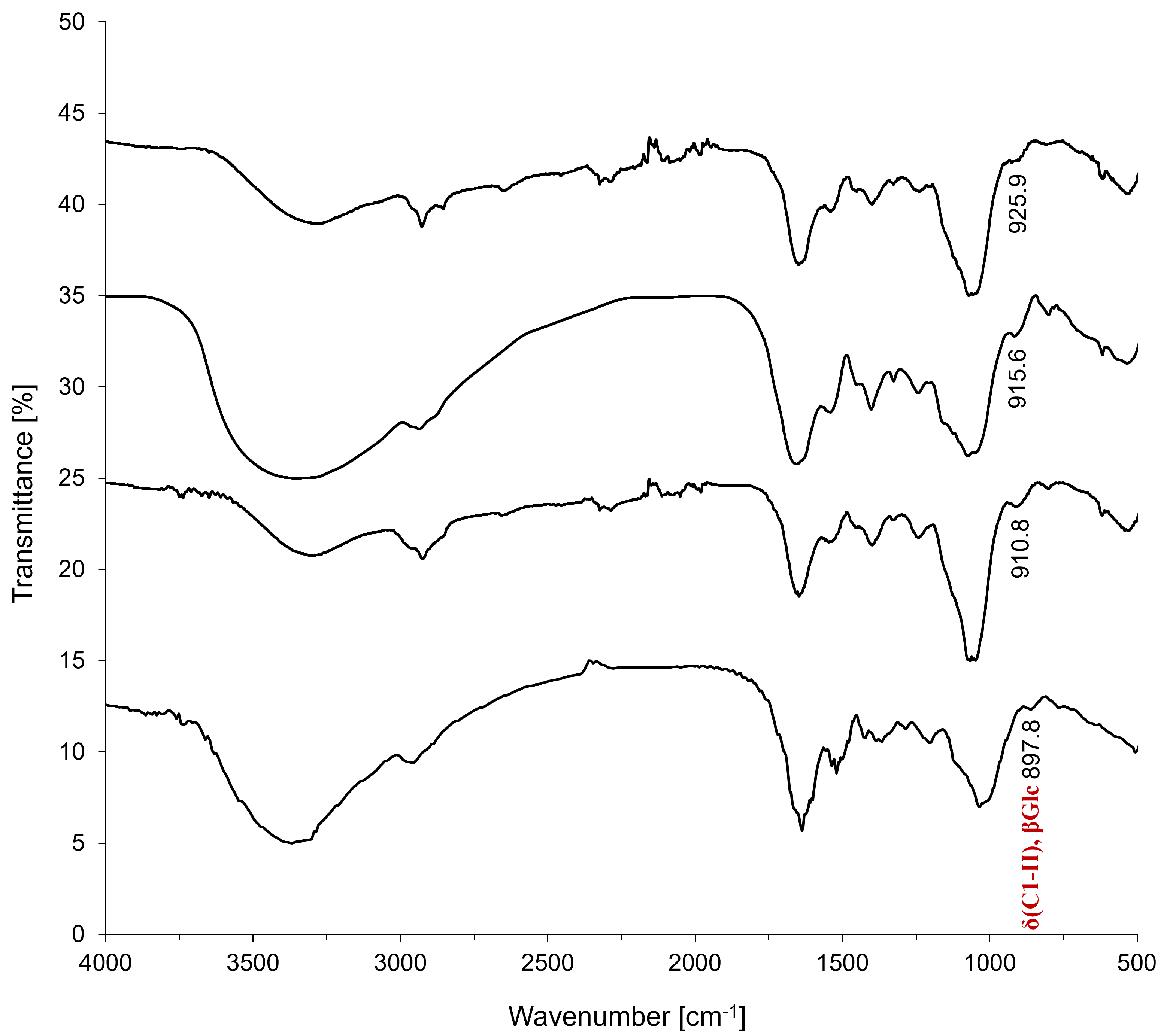

Supplement: Supplementary file 1 [file polymers-17-00719-s001.zip › Figure S3.tif]
